# Conspecific olfactory preferences and interspecific divergence in odor cues in a chickadee hybrid zone

**DOI:** 10.1002/ece3.5497

**Published:** 2019-08-01

**Authors:** Alex Van Huynh, Amber M. Rice

**Affiliations:** ^1^ Department of Biological Sciences Lehigh University Bethlehem PA USA

**Keywords:** black‐capped chickadee, Carolina chickadee, hybridization, olfaction, premating reproductive isolation, speciation

## Abstract

Understanding how mating cues promote reproductive isolation upon secondary contact is important in describing the speciation process in animals. Divergent chemical cues have been shown to act in reproductive isolation across many animal taxa. However, such cues have been overlooked in avian speciation, particularly in passerines, in favor of more traditional signals such as song and plumage. Here, we aim to test the potential for odor to act as a mate choice cue, and therefore contribute to premating reproductive isolation between the black‐capped (*Poecile atricapillus*) and Carolina chickadee (*P. carolinensis*) in eastern Pennsylvania hybrid zone populations. Using gas chromatography–mass spectrometry, we document significant species differences in uropygial gland oil chemistry, especially in the ratio of ester to nonester compounds. We also show significant preferences for conspecific over heterospecific odor cues in wild chickadees using a Y‐maze design. Our results suggest that odor may be an overlooked but important mating cue in these chickadees, potentially promoting premating reproductive isolation. We further discuss several promising avenues for future research in songbird olfactory communication and speciation.

## 1 INTRODUCTION

A central focus in evolutionary biology is to understand how species originate and how existing species boundaries are maintained. Natural hybridization—when separate species come into contact and mate—can provide important insights into the speciation process (Abbott et al., 2013). In animals, behavioral isolation (i.e., premating isolation) can act to maintain species boundaries upon secondary contact (reviewed in Servedio & Noor, 2003). Chemical communication in particular has been shown to act as an important premating reproductive isolating barrier in a wide variety of animal taxa (Smadja & Butlin, 2009) including insects (Coyne, Crittenden, & Mah, 1994; Sasakawa & Kon, 2018), fish (Kodric‐Brown & Strecker, 2001; Kozak, Head, & Boughman, 2011), reptiles (Barbosa, Font, Desfilis, & Carretero, 2006), amphibians (Dawley, 1984), and mammals (Johnston, 2003). However, conspecific preferences for interspecific odor cues have never been documented in a natural songbird hybrid zone (Caro, Balthazart, & Bonadonna, 2015).

Songbird hybrid zones have provided many insights into how behavioral isolation maintains species boundaries upon secondary contact (Edwards et al., 2005). While visual signals (Bleiweiss, 2004; Moller & Cuervo, 1998; Patten, Rotenberry, & Zuk, 2004; Sætre et al., 1997) and auditory signals (Haavie et al., 2004; Irwin, 2000; King, West, & Eastzer, 1980; Patten et al., 2004; Slabbekoorn & Smith, 2002) have been shown to act as premating barriers between hybridizing songbird species, such a role for olfaction has not been documented. However, songbirds do possess a working olfactory system (reviewed in Balthazart & Taziaux, 2010), including olfactory bulbs (Bang & Cobb, 1968), olfactory receptors (Steiger, Fidler, Valcu, & Kempenaers, 2008; Steiger, Kuryshev, Stensmyr, Kempenaers, & Mueller, 2009), and the ability to detect odor cues (Clark, Avilova, & Bean, 1993). Olfactory information for songbirds is most likely carried in uropygial gland oils, which have been shown to possess species‐specific chemical compositions (Soini, Whittaker, Wiesler, Ketterson, & Novotny, 2013). Recent work suggests a role for olfaction in many aspects of avian ecology (reviewed in Caro & Balthazart, 2012) including species discrimination (Bonnadonna & Mardon, 2010; Whittaker et al., 2011; Zhang, Du, & Zhang, 2013), sex discrimination (Amo et al., 2012; Soini et al., 2007; Whittaker et al., 2010; Zhang, Sun, & Zuo, 2009; Zhang, Wei, Zhang, & Yang, 2010), threat detection (Amo, Galván, Tomás, & Sanz, 2008; Amo, Visser, & Oers, 2011; Roth, Cox, & Lima, 2008), individual quality (Amo et al., 2012; Whittaker, Gerlach, Soini, Novotny, & Ketterson, 2013), aggression (Whittaker et al., 2018), nest recognition (Caspers, Hoffman, Kohlmeier, Krüger, & Krause, 2013; Golüke, Dörrenberg, Krause, & Caspers, 2016), and kin recognition (Bonadonna & Sanz‐Aguilar, 2012; Caspers, Gagliardo, & Krause, 2015; Caspers et al., 2017; Coffin, Watters, & Mateo, 2011; Krause, Kruger, Kohlmeier, & Caspers, 2012). Still, whether conspecific preferences for divergent odor cues exist in natural songbird hybrid zones and therefore whether odor cues might function as a premating barrier in songbirds remain unknown (Campagna, Mardon, Celerier, & Bonadonna, 2011; Caro et al., 2015). Here, we explore the potential for olfactory communication to act in premating reproductive isolation between the black‐capped and Carolina chickadee.

**Figure 1:**
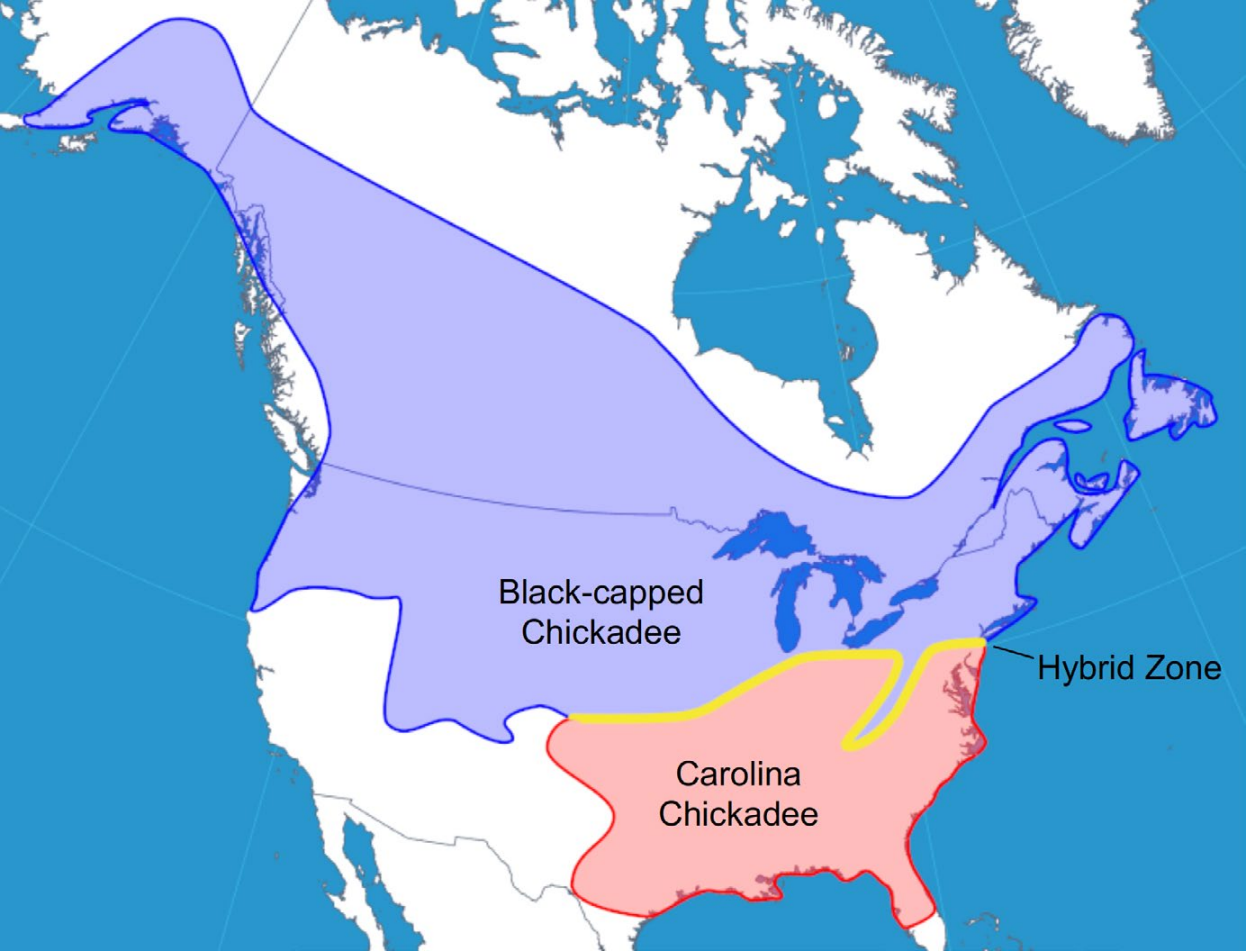
Range map of the black‐capped chickadee, Carolina chickadee, and approximate location of their hybrid zone

The black‐capped (*Poecile atricappilus*) and Carolina chickadee (*P. carolinensis*) are sister taxa (Harris, Carling, & Lovette, 2014) that occupy parapatric ranges in North America. The black‐capped chickadee resides in the northern half of the United States and in the southern half of Canada, while the Carolina chickadee occupies a range in the southeastern United States (Figure 1; McQuillan & Rice, 2015). The ranges of these two species overlap in a long but very narrow hybrid zone that stretches from New Jersey to Kansas, which is moving northward due to climate change (Figure 1; Taylor, Curry, White, Ferretti, & Lovette, 2014; Taylor, White, et al., 2014). Within this region of sympatry, the two species are able to successfully hybridize. However, postzygotic reproductive barriers are present. Similar to findings from other parts of the hybrid zone (Bronson, Grubb, Sattler, & Braun, 2005), breeding data collected from our hybrid zone transect in southeastern Pennsylvania shows that eggs produced by mixed‐species parental pairs are less likely to hatch compared to eggs produced by conspecific pairings (Figure S1). Additional costs to hybridization are also found in adult birds of mixed ancestry. Hybrid chickadees are compromised in their spatial memory ability (McQuillan, Roth, Huynh, & Rice, 2018), which is important for fitness in scatter‐hoarding species such as chickadees (Sonnenberg, Branch, Pitera, Bridge, & Pravosudov, 2019). Hybrid chickadees also are less likely to solve novel problems (McQuillan et al., 2018). Taken together, the reduced hatching success of hybrid offspring combined with cognitive deficiencies in adult hybrids suggests there are severe costs to hybridization for chickadees in our eastern Pennsylvania hybrid zone populations. Further, within the hybrid zone, the black‐capped and Carolina chickadee possess similar plumage (Robbins, Braun, & Tobey, 1986) and each species can learn the song of the other (Kroodsma, Albano, Houlihan, & Wells, 1995). Therefore, these two signals commonly implicated in behavioral isolation in songbirds may not be reliable indicators of species identity in this chickadee hybrid zone. We therefore sought to test two requirements for olfactory signaling to function in premating reproductive isolation between currently hybridizing black‐capped and Carolina chickadees: (1) the production of species‐specific chemical compounds and (2) conspecific odor preferences.

Here, we report chemical differences in wild‐caught hybrid zone black‐capped and Carolina chickadee uropygial oils. Uropygial oils are a main source of odor cues in birds, the chemical composition of which is known to be species‐specific across many songbird species (Soini et al., 2013). We also show that hybrid zone birds of both species show preferences for conspecific whole‐body odor cues over heterospecific whole‐body odor cues, suggesting a possible role for olfaction in reproductive isolation.

## 2 METHODS

### 2.1 Field methods and animal housing

We captured wild chickadees at five geographically proximate sites within the hybrid zone in eastern Pennsylvania (total transect length 30 km; Nockamixon State Park 40°26′10.7″N, 75°14′39.0″W, DeSales University 40°32′41.7″N, 75°22′29.5″W, Lehigh University 40°36′5.2″N, 75°21′34.1″W; Jacobsburg State Park 40°47′4.0″N, 75°17′34.7″W; Bangor 40^o^53′42.8″N, 75^o^10′32.0″W). Birds were caught using mist nets at feeders or by using song playback in conjunction with a clay chickadee model. Upon capture, we banded, measured, and weighed each bird. A small blood sample was collected for ancestry (McQuillan, Huynh, Taylor, & Rice, 2017) and sex determination (Griffiths, Double, Orr, & Dawson, 1998). All procedures were approved by Lehigh University's Institutional Animal Care and Use Committee (Protocol #215). Animal capture and transport were conducted under the U.S. Geological Survey federal bird banding permit 23810, U.S. Fish and Wildlife Service permit MB69567A‐0, Pennsylvania Game Commission permits 103 and 145, and Pennsylvania Bureau of State Parks permit 2016–18.

For the chemical analysis of uropygial oils, we sampled 41 black‐capped and 40 Carolina adult chickadees between December 2015 and December 2016. The uropygial gland of these birds was cleaned with 75% ethanol and gently squeezed with sterile forceps. A small sample of oil (~1 mg) was collected in the tip of a capillary tube. For the behavioral tests of odor preference, we captured 20 black‐capped males, 9 black‐capped females, 20 Carolina males, and 10 Carolina females between February 2017 and November 2018 and transported them by car to an outdoor aviary at Lehigh University. The difference in capture rate between the sexes could be due to a number of factors, including sex differences in attraction to our model and song playback. No birds were tested or held in captivity during the breeding season (mid‐March through July). Birds were housed individually in 0.46 m × 0.61 m × 0.61 m cages during their testing period and were visually but not aurally isolated from one another. During this time, all birds were sustained on an ad libitum diet of sunflower seeds, pine nuts, and water containing a vitamin supplement, as well as 15 waxworms and 20 mealworms per each day.

### 2.2 Genetic determination of species ancestry

Because black‐capped, Carolina, and hybrid chickadees are morphologically similar and song is not a reliable species identifier within the hybrid zone (Kroodsma et al., 1995), we utilized genetic markers to assign ancestry to each bird (McQuillan et al., 2017, 2018). Briefly, genomic DNA was extracted from blood samples using a Qiagen DNeasy blood and tissue kit (QIAGEN). We genotyped all birds at 10 species‐diagnostic single nucleotide polymorphism markers (McQuillan et al., 2017). We used STRUCTURE (Hubisz, Falush, Stephens, & Pritchard, 2009) to estimate admixture proportions and assign ancestry categories for each bird. To do this, we combined the genotypes of our test subjects with a larger dataset from multiple Pennsylvania hybrid‐zone populations, as well as known pure‐species individuals from allopatric populations of both species (New York and Louisiana, USA). We ran STRUCTURE on this larger dataset of over 400 total genotypes using the same program settings as McQuillan et al. (2017, 2018). Following McQuillan et al. (2018), birds with admixture values within the average 90% credible interval of known pure individuals were classified as either a pure black‐capped or Carolina individual. In contrast, birds with admixture values outside of the average 90% credible interval for known parentals were classified as hybrids and were not used in this study.

### 2.3 Uropygial oil collection and GC‐MS

Oil samples were extracted in 300 μl of dichloromethane overnight at 4°C. Chemical analysis was performed using gas chromatography–mass spectrometry (Shimadzu QP 2010 Ultra GC‐MS equipped with a SHRX1‐5US column; 30 m length, 0.25 mm thickness). Samples were run using conditions adapted from Zhang et al. (2013). Runs were performed in splitless mode on 3 μl of sample at a linear ramp of 70°C to 280°C over 42 min with a final hold at 280°C for 15 min (column pressure, 31.6 psi; total flow, 101.3 μl/min; column flow 3.86 ml/min; linear velocity 72 cm/s). We measured the relative abundance or total proportion of each compound by integrating the area under each peak and converting this area into a percentage of the total gas chromatograph area.

### 2.4 Odor preference trials

**Figure 2:**
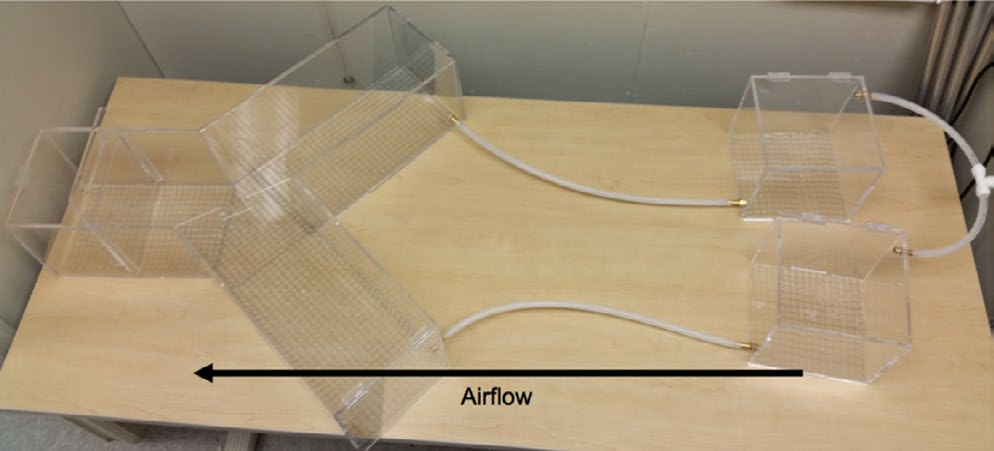
Y‐maze odor preference chamber (left) and odor donor chambers (right). During preference tests, an opaque screen prevents birds in the Y‐maze from seeing birds in the odor donor chambers. The odor donor chambers are kept in darkness during the preference trials to keep the odor donor birds still and silent. Perches are not shown in photo (see main text)

We tested males and females of both species for their odor preferences. We chose to test preferences in both sexes because in species showing biparental care, as is the case in chickadees, both female and male preferences have been shown to be evolutionarily significant (Edward & Chapman, 2011). All birds were subjected to two sequential no‐choice preference trials in a Y‐maze chamber (Figure 2; height: 20 cm, choice arms: 45 cm, starting arm: 30 cm, width of choice arms and starting arm: 20 cm). While both sequential no‐choice trials and two‐choice trials allow a measure of which odor individuals prefer (Dougherty & Shuker, 2015), we used no‐choice trials because they additionally provide information on the absolute preference of each odor individually. Absolute preferences for odors can have biologically important implications, such as providing insight into whether heterospecific odors are actively avoided. Our Y‐maze contained two wooden perches in each of the choice arms and two wooden perches in the starting arm. Each bird was acclimated once to the chamber for one hour with no odor source 3 days after capture with food supplied throughout the chamber to promote exploration. The first no‐choice test was conducted 3 days after successful acclimation, and the second no‐choice test was conducted 3 days after the first test. During each no‐choice test, air flow was supplied to both arms of the Y‐maze so that air could faintly be felt at the ends of the arms. However, one arm contained an odor source from either a live conspecific or heterospecific bird of the opposite sex, which was randomly assigned to one of the two odor donor chambers (Figure 2; 20 cm × 20 cm × 20 cm). The order of these two tests (conspecific vs. heterospecific odor donor) was also determined randomly.

During each trial, the Y‐maze and the odor donor chambers were separated visually by an opaque cardboard divider and the lights in the room were turned off. Two standing lamps were used to illuminate the area of the room with the Y‐maze, while the odor donor chambers were confined to darkness so that the odor donor birds remained still and silent. At the beginning of each trial, the test bird was confined to the starting area and allowed to acclimate for 5 min before being released into the Y‐maze. Upon release, all birds were recorded in the Y‐maze with a video camera. We designated the start of a 15‐min testing period as soon as the bird had experienced both sides of the Y‐maze (i.e., when bird entered the arm of the Y‐maze opposite its initial choice after release). Birds were designated as nonparticipatory if they did not explore both arms of the Y‐maze within 30 min and were removed from the study (three birds in total were removed; final black‐capped male *n* = 19; final black‐capped female *n* = 9; final Carolina male *n* = 17; final Carolina female *n* = 10). The time spent by the bird in the odor arm of the Y‐maze during the 15 min testing period was analyzed from the videos of these trials. The bird was considered to be investigating the odor arm when it was on or beyond the wooden perch in the odor arm most proximal to the center of the Y‐maze. All testing apparatuses were cleaned with ethanol and allowed to air dry between trials.

In our Y‐maze design, we used live birds as odor sources; we therefore tested preferences for whole‐body odor cues as opposed to odors from only uropygial oil secretions. The chemical composition of uropygial oil secretions and chemicals extracted directly from feathers have been shown to differ (Sandilands, Powell, Keeling, & Savory, 2004; Zhang et al., 2013). Results from whole‐body odor preference tests may be more ecologically relevant to how these birds encounter odor cues in the wild, as this method incorporates all odors and not just those derived from the uropygial gland.

### 2.5 Statistical analysis

#### 2.5.1 Uropygial oils

To correct for the nonindependency of our proportion data, we used an empirical logit transformation by taking the natural logarithm of *p* + *ε*/(1−*p* + *ε*), where *p* is the proportion of that compound in the entire sample and ε is the minimum nonzero proportion of the dataset, that is, 0.01 (Amo et al., 2012; Armitage, Berry, & Matthews, 1994; Baum, 2008; Warton & Hui, 2011; Whittaker et al., 2010; Zhang et al., 2013). To test for differences between black‐capped and Carolina chickadees in uropygial oil chemical profiles, we first conducted a principal components analysis (PCA) on the transformed gas chromatograph data (*N* = 81) to reduce the dataset of 146 different compounds into a smaller number of principal components (PCs). PCA is the most common method used to analyze avian preen oil chemistry (Amo et al., 2012; Fischer, Haliński, Meissner, Stepnowski, & Knitter, 2017; Leclaire et al., 2012; Lopez‐Perea & Mateo, 2019; Shaw, Rutter, Austin, Garvin, & Whelan, 2011; Tuttle et al., 2014; Whittaker et al., 2013; Whittaker et al., 2019; Whittaker et al., 2018; Whittaker et al., 2010; Zhang et al., 2013) and it allows an examination of which factors best explain the main axes of variation in our data, represented by PCs. We used a multivariate analysis of variance (MANOVA) on nontrivial principal components selected based on comparing a scree plot of the principal components with a broken‐stick distribution (Jackson, 1993), using species, date, sex, and all interactions as fixed factors.

We then used type‐II ANOVAs and least square means comparisons to further analyze individual PCs. Examining individual PCs in this way allowed us to look for species differences, while controlling for the effects of sampling date and the potential interaction effects of sampling date with other factors. This is particularly important concerning our data, since we collected preen oil samples throughout the calendar year and significant seasonal variation in preen oil chemistry has been observed in other species (Reneerkens, Piersma, & Damsté, 2002, 2005; Soini et al., 2007; Whelan, Levin, Owen, & Garvin 2010; Whittaker et al., 2019).

To analyze models of each individual PC, we performed a step‐wise model simplification procedure by removing the least significant variable, starting with higher order interactions; the initial full model contained species, date, sex, and all interactions as fixed factors. After removing a variable, we performed a likelihood ratio test (LRT) to assess the predictive effect of the focal term. If the newly simplified model explained significantly less variation in the response variable, then the focal term was retained. We repeated this process until we were left with a final model containing only those fixed effects that were significant predictors. We further validated our final model fits by comparing corrected Akaike information criterion (AICc) scores across all models using the R package *AICcmodavg* (Mazerolle, 2017). We chose to use a model reduction approach because we did not know a priori what variation in the data our PCs represent in terms of the effects of species, date, sex, and their interactions. Therefore, the elimination of nonsignificant factors from our models is appropriate, since their effects may not be captured by a given PC. Final best‐fit models were evaluated with type‐II ANOVAs using the R package *car* (Fox, 2007). Significant species effects were further compared by calculating the least square means, which corrects for other main effects, using the R package *lsmeans* and the “pairs” function (Lenth, 2016).

Rotation values of the first PC (35% of the total variance in compound relative abundances) were negatively correlated with earlier eluting compounds and positively correlated with later eluting wax‐ester compounds (Table S1). To validate the interpretation of PC1, we also manually calculated the ratio of the relative abundances of ester to nonester compounds for each individual as an alternate response variable. Model simplification for this analysis was conducted using the same LRT and AICc methods used to model our principal components. As described above, we evaluated the best‐fit model with a type‐II ANOVA and least square means comparisons. All analyses were conducted in R [3.5.2] (R Core Team, 2018).

#### 2.5.2 Odor preference trials

Because of the small sample sizes of each group (black‐capped male *n* = 19; black‐capped female *n* = 9; Carolina male *n* = 17; Carolina female *n* = 10), we tested the hypothesis that conspecific odors are preferred over heterospecific odors by using paired one‐tailed nonparametric Wilcoxon rank‐sum tests to compare time spent by chickadees with conspecific versus heterospecific odors. We also tested whether odor preferences differed from the random expectation for time spent in the choice arm of the Y‐maze (ratio of choice area volume/total Y‐maze volume × test duration of 900 s = 252 s) using one‐sample Wilcoxon rank‐sum tests. To test whether the strength of conspecific odor preferences varied throughout the year, we fit a linear model fit of the time spent with the conspecific odor by the fixed factors of Julian day, species, and sex. All analyses were conducted in R [3.5.2] (R Core Team, 2018).

## 3 RESULTS

### 3.1 Uropygial oils

The gas chromatograms of our chickadee oil samples revealed on average 34 (±22) compounds in a single individual's oil sample. Compound class identification using a NIST08 mass spec library predicted linear and branched alkanes eluting prior to ~35 min and ester compounds eluting after ~35 min.

We selected the first 4 principal components based on the scree plot of our analysis (Figure S2), which together explained 64% of the variation in the total relative abundances of all compounds (35%, 14%, 8%, and 7%, respectively). These principal components differed significantly by species (MANOVA, *F* = 10.41, *p* < .001) and by date (MANOVA, *F* = 43.66, *p* < .001). We also found a significant interaction between species and date (MANOVA, *F* = 2.64, *p* < .05). While the two sexes did not differ significantly in PC values (MANOVA, *F* = 0.81, *p* = .52), there was a significant three‐way interaction of species, date, and sex (MANOVA, *F* = 2.91, *p* < .05).

Our best‐fit model for PC1 contained only species and date as fixed factors (Table 1). PC1 values were significantly different between the two species and varied by date (Table 2), with black‐capped chickadees showing significantly higher PC1 values than Carolina chickadees (LSmeans contrast *t*‐ratio = 2.00, *p* < .05). Based on its rotation values, PC1 was negatively correlated with earlier eluting alkane compounds and positively correlated with later eluting ester compounds (Table S1). Our best‐fit model for the ratio of ester to nonester compounds included the three main effects of species, date, and sex as well as the interaction terms of species × sex and date × sex (Table 1). Corresponding with the species differences in PC1, the ratio of ester to nonester compounds was significantly greater in black‐capped chickadees than in Carolina chickadees (Table 2, LSmeans contrast *t*‐ratio = 3.13, *p* < .01). Although the effect of species changes depending on sex as indicated by the significant species x sex two‐way interaction (Table 2), a plot of the interaction shows that it does not preclude our ability to interpret the simple main effect of species (Figure S3). Our best‐fit model for PC2 was the full model with species, date, sex, and all interactions as fixed factors (Table 1). PC2 showed a more complicated relationship with a significant main effect of species, significant two‐way interactions of species × date and species × sex, as well as a significant three‐way interaction of species, date, and sex (Table 2). As shown by an interaction plot, the significant influence of sex on species differences did not preclude our ability to interpret the simple main effect of species (Figure S4). Nominalizing date to before, during, and after the breeding season revealed species differences in PC2 during the breeding season period (Figure S5). Because of this interaction, we restricted our analysis of PC2 to individuals caught only during the breeding season and found that black‐capped chickadees have significantly higher PC2 values (LSmeans contrast *t*‐ratio = 3.31, *p* < .01). However, the two species still show a similar significant difference in PC2 even without this restriction (LSmeans contrast *t*‐ratio = 3.43, *p* < .01). There were no significant species differences in PCs 3 and 4.

**Table 1:** Model selection based on LRT and AICc for PC1, PC2, and the ratio of ester to nonester compounds

Model	LRT	AICc
*PC1*		
Full model		365.2
Species, date, sex	.379	359.9
**Species, date**	**.631**	**357.8**
Species	<.001	393.2
Date	.046	359.8
*PC2*		
**Full model**		**321.1**
Species, date, sex, species × date, species × sex, date × sex	.004	327.2
*Ratio of ester to nonester compounds*		
Full model		831.0
Species, date, sex, species × date, species × sex, date × sex	.119	831.1
Species, date, sex, species × date, species × sex	.002	838.7
Species, date, sex, species × date, date × sex	.033	833.4
**Species, date, sex, species **×** sex, date **×** sex**	**.924**	**828.5**

Note

LRT *p*‐values are for the specified model compared to the last model above it that passed the LRT (*p*‐value > .05). Best‐fit models are highlighted in bold.

**Table 2:** Analysis of variance (type‐II tests) of PC1 (Adjusted *R* ^2^ = .41, *F*_2,78_ = 28.37, *p* < .001), PC2 (Adjusted *R* ^2^ = .27, *F*_7,64_ = 4.737, *p* < .001), and the ratio of ester to nonester compounds (Adjusted *R* ^2^ = .23, *F*_5,66_ = 5.32, *p* < .001)

	SS	*df*	*F*‐value	*p*‐Value
*PC1*				
Species	32.34	1	3.991	**.049**
Date	420.53	1	51.907	**<.001**
Residual	631.92	78		
*PC2*				
Species	28.266	2	3.319	**.043**
Date	0.196	1	0.046	.831
Species × date	60.929	1	14.308	**<.001**
Species × sex	19.192	1	4.507	**.038**
Date × sex	12.611	1	2.961	**.009**
Species × date × sex	35.309	1	8.291	**.005**
Residual	272.545	64		
*Ratio of ester to nonester compounds*				
Species	56,367	1	11.043	**.001**
Date	50,151	1	9.825	**.003**
Sex	2,773	1	0.543	.464
Species × sex	25,935	1	5.081	**.028**
Date × sex	55,554	1	10.884	**.002**
Residual	336,883	66		

Note

Significant *p*‐values are highlighted in bold.

#### 3.1.1 Odor preference trials

**Figure 3:**
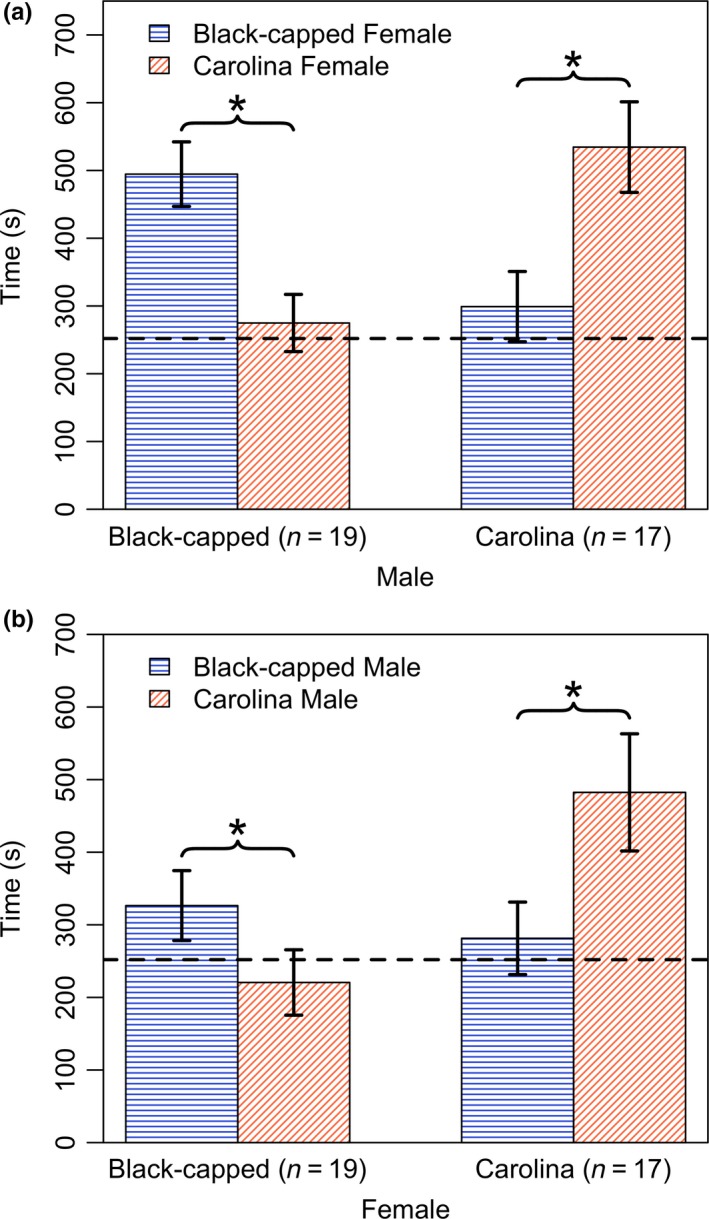
Time spent with the odor arm of the Y‐maze across two no‐choice preference tests for (a) males and (b) females of both species. Time spent with black‐capped odors is shown in bars with blue horizontal lines, and time spent with Carolina odors is shown in bars with red diagonal lines. The dashed line indicates the random expectation for time in the odor arm

Male birds of both species showed significant preferences for conspecific female odors over heterospecific female odors (Figure 3a; black‐capped male *V* = 163, *p* < .01; Carolina male *V* = 132, *p* < .01). Time spent by males with conspecific female odor was significantly higher than expected for random movement (Figure 3a; black‐capped male *V* = 180, *p* < .001; Carolina male *V* = 136, *p* < .01), while the time spent with heterospecific female odor was not different from random movement (Figure 3a; black‐capped male *V* = 102, *p* = .8; Carolina male *V* = 88, *p* = .6). Likewise, both groups of female birds also showed significant preferences for conspecific male odors over heterospecific female odors (Figure 3b; black‐capped female *V* = 39, *p* < .05; Carolina female *V* = 47, *p* < .05). Carolina females spent significantly more time with conspecific odors than expected for random movement (Figure 3b; *V* = 51, *p* < .05), but not with heterospecific odors (Figure 3b; *V* = 34, *p* = .5) However, black‐capped female preferences for either male odor did not differ significantly from random movement (Figure 3b; for black‐capped male odor *V* = 34.5, *p* = .17; for Carolina male odor *V* = 16, *p* = .5). Our data showed no apparent seasonal effect on odor preferences throughout the year in both sexes of either species (Figure S6).

## 4 DISCUSSION

Overall, our results are consistent with a possible role for olfactory signaling in premating reproductive isolation in chickadees. Within the hybrid zone, these two species show differences in their uropygial oil chemistry (Table 2) as well as significant preferences for conspecific bird odors over those of heterospecific birds (Figure 3). To our knowledge, this is the first evidence of olfaction‐based species discrimination in a natural songbird hybrid zone.

Reproductive isolation due to divergent olfactory signals and preferences is known to occur in insects (Löfstedt et al., 1986; Sasakawa & Kon, 2018; Singer, 1998), as well as in many vertebrate taxa including fish (Kozak et al., 2011; McLennan & Ryan, 1999; Rafferty & Boughman, 2006), amphibians (Dawley, 1984), reptiles (Barbosa et al., 2006), and mammals (Christophe & Baudoin, 1998). While discrimination between conspecific and heterospecific odors has been observed in several songbird systems, conspecific odor preference within naturally hybridizing songbird species has not been previously documented. For example, female waxwings were found to prefer odor cues of their own species over cues of their sympatric sibling species. However, because these two species do not currently engage in hybridization, it is unclear whether these preferences played a role in the actual speciation process (Zhang et al., 2013). Likewise, crimson rosellas have been found to discriminate between odors of related subspecies based on the latency for birds to enter their nest boxes when presented with odor cues (Mihailova, Berg, Buchanan, & Bennett, 2014), but these experiments did not test birds from their natural hybrid zone and direct preferences for odor cues were not measured. Additionally, while odor discrimination was found in juncos (Whittaker et al., 2011) and zebra finches (Krause et al., 2014), the heterospecifics used in these preference trials do not naturally hybridize with the focal species.

We found significant species differences in the principal component description of uropygial oil profiles of black‐capped and Carolina chickadees, including differences in the ratio of wax‐ester to nonester compounds (Table 2). Similar relative abundance ratios of compound mixtures are enough to encode communicatory information in mammals (Sun & Muller‐Schwarze, 1998a, 1998b), insects (Byers & Struble, 1990; Coyne et al., 1994; Singer, 1998; Wang, Zhao, & Wang, 2005), and birds (Zhang et al., 2013) and shifts in wax‐ester ratios have been found to coordinate with breeding in the Scolopacidae (Reneerkens, Piersma, & Damsté, 2002, 2005). Further, a number of studies show support for a genetic basis of uropygial oil chemistry in birds (Leclaire et al., 2012; Soini et al., 2013; Whittaker et al., 2010). Thus, chemical differences in songbird uropygial oils such as those found here could function in mate choice and reproductive isolation by carrying information on species identity.

Although little is known about the biosynthesis of uropygial oils, hormone levels (Whittaker et al., 2018), diet (Apandi & Edwards, 1964), and age (Sandilands et al., 2004) have all been shown to have effects on oil composition. Promising avenues of future research could address the possibility of uropygial oils acting as an honest signal of mate quality (McGlothlin et al., 2008; Velando, Beamonte‐Barrientos, & Torres, 2006). For example, oil composition could carry information on hormone levels, which are known to be closely tied to cognitive ability (reviewed in Healy & Hurly, 2004) and aggression (Whittaker et al., 2018), both of which are important for fitness in chickadees (Bronson, Grubb, Sattler, & Braun, 2003; Sonnenberg et al., 2019). Additionally, whether or not these cues could potentially act as a “magic trait” during speciation with gene flow could be considered. Magic traits are those that are under divergent selection while also promoting assortative mating in sympatry (Servedio, Doorn, Kopp, Frame, & Nosil, 2011). Well‐studied examples of magic traits include body size (Nagel & Schluter, 1998), body shape (Langerhans, Gifford, & Joseph, 2007), beak morphology and song (Podos, 2001), coloration (Reynolds & Fitzpatrick, 2007), and diet (Snowberg & Bolnick, 2008). If uropygial oil biosynthesis is affected by diet, then these oil‐derived odor cues could serve as an indicator of resource specialization, potentially contributing to assortative mating within populations undergoing divergent adaptation. Further research on the factors affecting the chemical composition of uropygial oils, and on the role of oil‐derived odor cues in the mate preferences of songbird populations in the early stages of divergence will be necessary to evaluate these possibilities.

Our experiments indicate a clear preference for conspecific whole‐body odors in both species of chickadees (Figure 3). These preferences were present in male as well as female birds. Male mating preferences, especially in species displaying biparental care, can be just as important as female choice (Johnstone, Reynolds, & Deutsch, 1996) and recent work has begun to acknowledge the potentially widespread role of male mate choice in animals (reviewed in Edward & Chapman, 2011). Male choice can act during the selection of a partner (Jones, Monaghan, & Nager, 2001), or postcopulation, such as in the allocation of parental care after males have the opportunity to assess female quality (Matessi, Carmagnani, Griggio, & Pilastro, 2008). Further, mate preference models demonstrate that the evolution of reproductive isolation and reinforcement can occur solely through male mating preferences (Servedio, 2007). Mate choice preference for female odor cues have been empirically shown in red‐spotted newts (Verrell, 1985), spiders (Gaskett, Herberstein, Downes, & Elgar, 2004), and more recently in *Drosophila*, where such preferences drive reproductive isolation (Shahandeh, Pischedda, & Turner, 2017). Our odor preference results are consistent with the possible role of male mate choice in reproductive isolation.

In our tests, time spent with heterospecifics did not differ from random expectation in all four groups of birds. This raises the possibility that selection could be acting to promote conspecific preferences instead of avoidance to heterospecific odors. Hybridization, although costly, could incur fitness advantages if a conspecific mate cannot be found, in which case, aversion to heterospecific odors could be maladaptive. Interestingly, female black‐capped chickadee preferences for conspecifics also did not differ from random expectation (Figure 3). This could indicate that odor cues are weighed differently between the sexes or species or that females may be incorporating additional types of information in their mate choice decisions. Bronson et al. (2003) found that both black‐capped and Carolina females show preferences for black‐capped males, but that this preference switches to Carolina males when they are allowed to observe male–male social interactions. Thus, mate choice may be more complex and context‐dependent in female chickadees. Research on mating systems indicates that multiple cues may act in songbird mate choice (Bro‐Jørgensen, 2009; Byers & Kroodsma, 2009; Candolin, 2003; Gil & Gahr, 2002; Hill, 2006; Otter & Ratcliffe, 1996; Patten et al., 2004; Whittaker & Gerlach, 2016). Emerging work contends that female birds show individual variation in their ability to perceive different modes of communication and that multimodal signaling is thus particularly important for advertising in males (Ronald, Fernández‐Juricic, & Lucas, 2018). We suggest that odor could be an overlooked signal that contributes in a multimodal fashion with more traditionally studied mating cues such as song, plumage, and social rank in chickadees and in songbirds generally.

Odor cues may function directly as a mate choice cue or indirectly through their effects on other aspects of songbird ecology. Chickadees overwinter in mixed‐species flocks, during which time they can often be found in close proximity to one another and their pair bonds are formed prior to the start of the breeding season, in late winter into the early spring (Odum, 1941). While interacting in close proximity within these winter flocks, birds may be able to incorporate olfactory cues into their mate choice decisions. Although our Y‐maze experimental design did not measure actual mate choice, which can differ from mate preferences depending on context (Yang, Blomenkamp, Dugas, Richards‐Zawacki, & Pröhl, 2019), it did allow us to isolate and measure preferences solely for odor cues in wild‐caught chickadees. Time spent with a potential mate or cue has been widely used as a proxy for mate choice (Bronson et al., 2003; Gaskett et al., 2004; Verrell, 1985; Yang, Richards‐Zawacki, Devar, & Dugas, 2016), and similar Y‐maze designs measuring time spent with odor cues have been used to assess odor preferences as a proxy for mate choice in songbirds (Bonadonna & Sanz‐Aguilar, 2012; Whittaker et al., 2011; Zhang et al., 2013). While not addressed in our experiments, odor may also play indirect roles in chickadee mate choice, for example, through male–male social interactions. Social rank is important in mate choice in chickadees (Bronson et al., 2003), and uropygial oil composition has been found to correlate with aggression in other songbirds (Whittaker et al., 2018). Thus, how chemical signaling could influence other aspects of songbird behavior indirectly related to mate choice such as male–male interactions is an interesting avenue for future research.

Whether olfactory preferences in songbirds are generally learned or innate is unknown. Learned mating preferences are well documented, affecting sexual isolation and the speciation process (reviewed in Verzijden et al., 2012). In other taxa, prenatal chemosensory learning has been demonstrated (Caubet, Jaisson, & Lenoir, 1992; Courtenay, 1989; Hepper & Waldman, 1992; Schaal & Orgeur, 1992) and sexual imprinting on diet‐based odor cues has been found to contribute to reproductive isolation (Delaney & Hoekstra, 2018; Kozak et al., 2011; Sasakawa & Kon, 2018). In several procellariform species, young chicks seem to demonstrate odor recognition (Cunningham, Buskirk, Bonadonna, Weimerskirch, & Nevitt, 2003; De Leon, Mínguez, & Belliure, 2003). Preference for extraneous odors has been found to develop during the incubation period in chickens (Sneddon, Hadden, & Hepper, 1998) while preferences for parental odors may be determined earlier in egg development in zebra finches (Caspers et al., 2017). Within the same family as chickadees (Paridae), blue tits have also been shown to exhibit odor recognition at the nestling stage (Rossi et al., 2017). While more work needs to be done in this area, the possibility that odor preferences are formed during development in chickadees could facilitate its function in reproductive isolation if individuals learn preferences from parental odors in pure‐species nests.

Although our results suggest that olfactory cues in this chickadee hybrid zone have the potential to contribute to assortative mating, it remains unknown whether direct selection against hybridization is driving the evolution of this premating isolation (i.e., reinforcement; Lewontin, 1974; Servedio & Noor, 2003). Postzygotic isolation in this chickadee hybrid zone has been documented in the form of reduced hatching success of interspecific breeding pairs (Figure S1, Bronson et al., 2005) and in the reduced cognitive abilities of adult hybrids (McQuillan et al., 2018). To test whether this maladaptive hybridization is directly reinforcing uropygial oil differences and odor preferences within the hybrid zone, further work should be conducted in allopatric populations. Away from the hybrid zone, where hybridization cannot occur, a reduction in conspecific preferences and oil differences may be observed—that is, a pattern of reproductive character displacement (Pfennig & Pfennig, 2012). Chemical cues have been shown to undergo reinforcement across multiple taxa in natural hybrid zones (reviewed in Smadja & Butlin, 2009), but this has not yet been observed in an avian system.

Work in other systems raises the interesting possibility that hybridization could affect olfactory preferences in songbirds. Compromised olfactory ability has been found in hybrid insects (Olsson et al., 2006) and skewed olfactory preference in hybrid individuals has been shown in mice (Christophe & Baudoin, 1998). The odors and odor preferences of hybrid chickadees are unknown, yet these traits could influence the evolution of populations along their hybrid zone by affecting general patterns of mate choice and introgression. Alternatively, if hybrid chickadees are somehow compromised in these traits, this could contribute to postzygotic isolation through their inability to advertise successfully or assess mate quality.

In conclusion, our results highlight the role that uropygial oils and odor cues may be playing in songbird mate choice and premating reproductive isolation along with more traditionally studied cues such as song and plumage characteristics. The differences in oil profiles combined with conspecific preferences in both species of chickadees suggest that olfaction could be acting directly in reproductive isolation in this hybrid system. Lastly, we argue that the developmental basis of songbird olfactory preferences, the possible reinforcement of these cues and preferences within the hybrid zone, and the chemical characteristics and preferences of hybrid individuals are promising avenues of future research.

## CONFLICT OF INTEREST

This paper is not under consideration for publication in any other journal. Both authors have read this manuscript, agreed to this submission, and have no conflicts of interest to disclose.

## AUTHOR CONTRIBUTIONS

A.V.H. conceived of the project idea, performed experiments, and analyzed data. A.V.H. and A.M.R. designed experiments and wrote the manuscript.

## Data Availability

All pertaining data files and R code can be found in the Dryad Digital Repository; https://doi.org/10.5061/dryad.7ht92p7.

## References

[ece35497-bib-0001] Abbott, R. , Albach, D. , Ansell, S. , Arntzen, J. W. , Baird, S. J. E. , Bierne, N. , … Zinner, D. (2013). Hybridization and speciation. Journal of Evolutionary Biology, 26(2), 229–246. 10.1111/j.1420-9101.2012.02599.x 23323997

[ece35497-bib-0002] Amo, L. , Avilés, J. M. , Parejo, D. , Peña, A. , Rodríguez, J. , & Tomás, G. (2012). Sex recognition by odour and variation in the uropygial gland secretion in starlings. Journal of Animal Ecology, 81(3), 605–613. 10.1111/j.1365-2656.2011.01940.x 22220811

[ece35497-bib-0003] Amo, L. , Galván, I. , Tomás, G. , & Sanz, J. J. (2008). Predator odour recognition and avoidance in a songbird. Functional Ecology, 22(2), 289–293. 10.1111/j.1365-2435.2007.01361.x

[ece35497-bib-0004] Amo, L. , Visser, M. E. , & van Oers, K. (2011). Smelling out predators is innate in birds. Ardea, 99(2), 177–184. 10.5253/078.099.0207

[ece35497-bib-0005] Apandi, M. , & Edwards, H. M. (1964). Studies on the composition of the secretions of the uropygial gland of some avian species. Poultry Science, 43(6), 1445–1462. 10.3382/ps.0431445

[ece35497-bib-0006] Armitage, P. , Berry, G. , & Matthews, J. N. S. (1994). Statistical methods in medical research. Hoboken, NJ: John Wiley and Sons.

[ece35497-bib-0007] Balthazart, J. , & Taziaux, M. (2010). The underestimated role of olfaction in avian reproduction? Behavioral Brain Research, 200(2), 248–259. 10.1016/j.bbr.2008.08.036 PMC2692081 18804490

[ece35497-bib-0008] Bang, B. G. , & Cobb, S. (1968). The size of the olfactory bulb in 108 species of birds. The Auk, 85, 55–61.

[ece35497-bib-0009] Barbosa, D. , Font, E. , Desfilis, E. , & Carretero, M. A. (2006). Chemically mediated species recognition in closely related *Podarcis* wall lizards. Journal of Chemical Ecology, 32(7), 1587–1598. 10.1007/s10886-006-9072-5 16718555

[ece35497-bib-0010] Baum, C. F. (2008). Stat tip 63: Modeling proportions. The Stata Journal, 8(2), 299–303.

[ece35497-bib-0011] Bleiweiss, R. (2004). Ultraviolet plumage reflectance distinguishes sibling bird species. Proceedings of the National Academy of Sciences of the United States of America, 101(47), 16561–16564. 15546987 10.1073/pnas.0406386101 PMC534516

[ece35497-bib-0012] Bonadonna, F. , & Sanz‐Aguilar, A. (2012). Kin recognition and inbreeding avoidance in wild birds: The first evidence for individual kin‐related odour recognition. Animal Behaviour, 84, 509–513. 10.1016/j.anbehav.2012.06.014

[ece35497-bib-0013] Bonnadonna, F. , & Mardon, J. (2010). One house two families: Petrel squatters get a sniff of low‐cost breeding opportunities. Ethology, 116, 176–182. 10.1111/j.1439-0310.2009.01725.x

[ece35497-bib-0014] Bro‐Jørgensen, J. (2009). Dynamics of multiple signaling systems: Animal communication in a world in flux. Trends in Ecology and Evolution, 25(5), 292–300. 20022401 10.1016/j.tree.2009.11.003

[ece35497-bib-0015] Bronson, C. L. , Grubb, T. C. , Sattler, G. D. , & Braun, M. J. (2003). Mate preference: A possible causal mechanism for a moving hybrid zone. Animal Behaviour, 65, 489–500. 10.1006/anbe.2003.2103

[ece35497-bib-0016] Bronson, C. L. , Grubb, T. C. , Sattler, G. D. , & Braun, M. J. (2005). Reproductive success across the black‐capped chickadee and Carolina chickadee hybrid zone in Ohio. The Auk, 122(3), 759–772.

[ece35497-bib-0017] Byers, B. E. , & Kroodsma, D. E. (2009). Female mate choice and songbird song repertoires. Animal Behaviour, 77, 13–22. 10.1016/j.anbehav.2008.10.003

[ece35497-bib-0018] Byers, J. R. , & Struble, D. L. (1990). Identification of sex‐pheromones of two sibling species in dingy cutworm complex, *Feltia jaculifera* (GN.) (Lepidoptera: Noctuidae). Journal of Chemical Ecology, 16, 2981–2992. 10.1007/BF00979489 24263270

[ece35497-bib-0019] Campagna, S. , Mardon, J. , Celerier, A. , & Bonadonna, F. (2011). Potential semiochemical molecules from birds: A practical and comprehensive compilation of the last 20 years studies. Chemical Senses, 37(1), 3–25. 10.1093/chemse/bjr067 21798850

[ece35497-bib-0020] Candolin, U. (2003). The use of multiple cues in mate choice. Biological Reviews, 78(4), 575–595. 10.1017/S1464793103006158 14700392

[ece35497-bib-0021] Caro, S. P. , & Balthazart, J. (2012). Pheromones in birds: Myth or reality? Journal of Comparative Physiology A, 196(10), 751–766. 10.1007/s00359-010-0534-4 PMC3522863 20490809

[ece35497-bib-0022] Caro, S. P. , Balthazart, J. , & Bonadonna, F. (2015). The perfume of reproduction in birds: Chemosignaling in avian social life. Hormones and Behavior, 68, 25–42. 10.1016/j.yhbeh.2014.06.001 24928570 PMC4263688

[ece35497-bib-0023] Caspers, B. A. , Gagliardo, A. , & Krause, E. T. (2015). Impact of kin odour on reproduction in zebra finches. Behavioral Ecology and Sociobiology, 69, 1827–1833. 10.1007/s00265-015-1995-9

[ece35497-bib-0024] Caspers, B. A. , Hagelin, J. C. , Paul, M. , Bock, S. , Willeke, S. , & Krause, E. T. (2017). Zebra finch chicks recognise parental scent, and retain chemosensory knowledge of their genetic mother, even after egg cross‐fostering. Scientific Reports, 7, 12859. 28993703 10.1038/s41598-017-13110-y PMC5634463

[ece35497-bib-0025] Caspers, B. A. , Hoffman, J. I. , Kohlmeier, P. , Krüger, O. , & Krause, E. T. (2013). Olfactory imprinting as a mechanism for nest odour recognition in zebra finches. Animal Behaviour, 86, 85–90. 10.1016/j.anbehav.2013.04.015

[ece35497-bib-0026] Caubet, Y. , Jaisson, P. , & Lenoir, A. (1992). Preimaginal induction of adult behavior in insects. The Quarterly Journal of Experimental Psychology B, 44(3–4), 165–178.

[ece35497-bib-0027] Christophe, N. , & Baudoin, C. (1998). Olfactory preferences in two strains of wild mice, *Mus musculus musculus* and *Mus musculus domesticus*, and their hybrids. Animal Behaviour, 56(2), 365–369. 10.1006/anbe.1998.0798 9787027

[ece35497-bib-0028] Clark, L. , Avilova, K. V. , & Bean, N. J. (1993). Odor thresholds in passerines. Comparative Biochemistry and Physiology A: Physiology, 104(2), 305–312. 10.1016/0300-9629(93)90322-U

[ece35497-bib-0029] Coffin, H. R. , Watters, J. V. , & Mateo, J. M. (2011). Odor‐based recognition of familiar and related conspecifics: A first test conducted on captive Humboldt penguins (*Spheniscus humboldti*). PLoS ONE, 6(9), e25002 10.1371/journal.pone.0025002 21957471 PMC3177858

[ece35497-bib-0030] Courtenay, S. C. (1989). Learning and memory of chemosensory stimuli by underyearling coho salmon *Oncorhynchus kisutch* (Walbaum). Doctoral dissertation, University of British Columbia.

[ece35497-bib-0031] Coyne, J. A. , Crittenden, A. P. , & Mah, K. (1994). Genetics of a pheromonal difference contributing to reproductive isolation in *Drosophila* . Science, 265, 1461–1464. 10.1126/science.8073292 8073292

[ece35497-bib-0032] Cunningham, G. B. , Van Buskirk, R. W. , Bonadonna, F. , Weimerskirch, H. , & Nevitt, G. A. (2003). A comparison of the olfactory abilities of three species of procellariiform chicks. Journal of Experimental Biology, 206, 1615–1620. 10.1242/jeb.00286 12682093

[ece35497-bib-0033] Dawley, E. M. (1984). Recognition of individual, sex and species odours by salamanders of the *Plethodon glutinosus*‐*P. jordani* complex. Animal Behavior, 32(2), 353–361. 10.1016/S0003-3472(84)80268-7

[ece35497-bib-0034] De Leon, A. , Mínguez, E. , & Belliure, B. (2003). Self‐odour recognition in European storm‐petrel chicks. Behaviour, 140(7), 925–933.

[ece35497-bib-0035] Delaney, E. K. , & Hoekstra, H. E. (2018). Diet‐based assortative mating through sexual imprinting. bioRxiv, 338848 10.1101/338848 PMC6854104 31844516

[ece35497-bib-0036] Dougherty, L. R. , & Shuker, D. M. (2015). The effect of experimental design on the measurement of mate choice: A meta‐analysis. Behavioral Ecology, 26(2), 311–319. 10.1093/beheco/aru125

[ece35497-bib-0037] Edward, D. A. , & Chapman, T. (2011). The evolution and significance of male mate choice. Trends in Ecology and Evolution, 26(12), 647–654. 10.1016/j.tree.2011.07.012 21890230

[ece35497-bib-0038] Edwards, S. V. , Kingan, S. B. , Calkins, J. D. , Balakrishnan, C. N. , Jennings, W. B. , Swanson, W. J. , & Sorenson, M. D. (2005). Speciation in birds: Genes, geography, and sexual selection. Proceedings of the National Academy of Sciences of the United States of America, 102, 6550–6557. 10.1073/pnas.0501846102 15851678 PMC1131863

[ece35497-bib-0039] Fischer, I. , Haliński, Ł. P. , Meissner, W. , Stepnowski, P. , & Knitter, M. (2017). Seasonal changes in the preen wax composition of the herring gull *Larus argentatus* . Chemoecology, 27, 127–139. 10.1007/s00049-017-0239-z 28804215 PMC5533864

[ece35497-bib-0040] Fox, J. (2007). “The car Package”. Vienna, Austria: R Foundation for Statistical Computing.

[ece35497-bib-0041] Gaskett, A. C. , Herberstein, M. E. , Downes, B. J. , & Elgar, M. A. (2004). Changes in male mate choice in a sexually cannibalistic orb‐web spider (Araneae: Araneidae). Behaviour, 141(10), 1197–1210. 10.1163/1568539042729676

[ece35497-bib-0042] Gil, D. , & Gahr, M. (2002). The honesty of bird song: Multiple constraints for multiple traits. Trends in Ecology and Evolution, 17(3), 133–141. 10.1016/S0169-5347(02)02410-2

[ece35497-bib-0043] Golüke, S. , Dörrenberg, S. , Krause, E. T. , & Caspers, B. A. (2016). Female zebra finches smell their eggs. PLoS ONE, 11(5) , e0155513 10.1371/journal.pone.0155513 27192061 PMC4871452

[ece35497-bib-0044] Griffiths, R. , Double, M. C. , Orr, K. , & Dawson, R. J. G. (1998). A DNA test to sex most birds. Molecular Ecology, 7, 1071–1075. 10.1046/j.1365-294x.1998.00389.x 9711866

[ece35497-bib-0045] Haavie, J. , Borge, T. , Bures, S. , Garamszegi, L. Z. , Lampe, H. M. , Moreno, J. , … Sætre, G. P. (2004). Flycatcher song in allopatry and sympatry – Convergence, divergence and reinforcement. Journal of Evolutionary Biology, 17(2), 227–237. 10.1111/j.1420-9101.2003.00682.x 15009256

[ece35497-bib-0046] Harris, R. B. , Carling, M. D. , & Lovette, I. J. (2014). The influence of sampling design on species tree inference: A new relationship for the new world chickadees (Aves: *Poecile*). Evolution, 68, 501–513. 24111665 10.1111/evo.12280

[ece35497-bib-0047] Healy, S. D. , & Hurly, T. A. (2004). Spatial learning and memory in birds. Brain, Behavior, and Evolution, 63, 211–220. 10.1159/000076782 15084814

[ece35497-bib-0048] Hepper, P. G. , & Waldman, B. (1992). Embryonic olfactory learning in frogs. The Quarterly Journal of Experimental Psychology B, 44(3–4), 179–197. 10.1080/02724999208250611 1598418

[ece35497-bib-0049] Hill, G. E. (2006). Female mate choice for ornamental coloration. Bird Coloration, 2, 137–200.

[ece35497-bib-0050] Hubisz, M. J. , Falush, D. , Stephens, M. , & Pritchard, J. K. (2009). Inferring weak population structure with the assistance of sample group information. Molecular Ecology Resources, 9, 1322–1332. 10.1111/j.1755-0998.2009.02591.x 21564903 PMC3518025

[ece35497-bib-0051] Irwin, D. E. (2000). Song variation in an avian ring species. Evolution, 54(3), 998–1010. 10.1111/j.0014-3820.2000.tb00099.x 10937272

[ece35497-bib-0052] Jackson, D. A. (1993). Stopping rules in principal components analysis: A comparison of heuristical and statistical approaches. Ecology, 74(8), 2204–2214. 10.2307/1939574

[ece35497-bib-0053] Johnston, R. E. (2003). The role of chemical communication in rodents: From pheromones to individual recognition. Journal of Mammalogy, 84, 1141–1162. 10.1644/BLe-010

[ece35497-bib-0054] Johnstone, R. A. , Reynolds, J. D. , & Deutsch, J. C. (1996). Mutual mate choice and sex differences in choosiness. Evolution, 50(4), 1382–1391. 10.1111/j.1558-5646.1996.tb03912.x 28565695

[ece35497-bib-0055] Jones, K. M. , Monaghan, P. , & Nager, R. G. (2001). Male mate choice and female fecundity in zebra finches. Animal Behaviour, 62(6), 1021–1026. 10.1006/anbe.2001.1843

[ece35497-bib-0056] King, A. P. , West, M. J. , & Eastzer, D. H. (1980). Song structure and song development as potential contributors to reproductive isolation in cowbirds (*Molothrus ater*). Journal of Comparative and Physiological Psychology, 94(6), 1028–1039. 10.1037/h0077737

[ece35497-bib-0057] Kodric‐Brown, A. , & Strecker, U. (2001). Responses of *Cyprinodon maya* and *C. labiosus* females to visual and olfactory cues of conspecific and heterospecific males. Biological Journal of the Linnean Society, 74, 541–548.

[ece35497-bib-0058] Kozak, G. M. , Head, M. L. , & Boughman, J. W. (2011). Sexual imprinting on ecologically divergent traits leads to sexual isolation in sticklebacks. Proceedings of the Royal Society B: Biological Sciences, 278(1718), 2604–2610. 10.1098/rspb.2010.2466 PMC3136826 21270044

[ece35497-bib-0059] Krause, E. T. , Brummel, C. , Kohlwey, S. , Baier, M. C. , Müller, C. , Bonadonna, F. , & Caspers, B. A. (2014). Differences in olfactory species recognition in the females of two Australian songbird species. Behavioral Ecology and Sociobiology, 68(11), 1819–1827. 10.1007/s00265-014-1791-y

[ece35497-bib-0060] Krause, E. T. , Kruger, O. , Kohlmeier, P. , & Caspers, B. A. (2012). Olfactory kin recognition in a songbird. Biological Letters, 8, 327–329. 10.1098/rsbl.2011.1093 PMC3367747 22219391

[ece35497-bib-0061] Kroodsma, D. E. , Albano, D. J. , Houlihan, P. W. , & Wells, J. A. (1995). Song development by black‐capped chickadees (*Parus Atricapillus*) and Carolina chickadees (*P. Carolinensis*). The Auk, 112(1), 29–43. 10.2307/4088764

[ece35497-bib-0062] Langerhans, R. B. , Gifford, M. E. , & Joseph, E. O. (2007). Ecological speciation in *Gambusia* fishes. Evolution, 61, 2056–2074. 10.1111/j.1558-5646.2007.00171.x 17767582

[ece35497-bib-0063] Leclaire, S. , Merkling, T. , Raynaud, C. , Mulard, H. , Bessiere, J. M. , Lhuillier, E. , … Danchin, E. (2012). Semiochemical compounds of preen secretion reflect genetic make‐up in a seabird species. Proceedings of the Royal Society B: Biological Sciences, 279, 1185–1193. 10.1098/rspb.2011.1611 PMC3267147 21937499

[ece35497-bib-0064] Lenth, R. V. (2016). Least‐squares means: The R package lsmeans. Journal of Statistical Software, 69(1), 1–33.

[ece35497-bib-0065] Lewontin, R. C. (1974). The genetic basis of evolutionary change. New York, NY: Columbia University Press.

[ece35497-bib-0066] Löfstedt, C. , Löfqvist, J. , Lanne, B. S. , Pers, J. N. C. V. D. , Hansson, B. S. , Lofstedt, C. , & Lofqvist, J. (1986). Pheromone dialects in European turnip moths *Agrotis segetum* . Oikos, 46, 250–257. 10.2307/3565474

[ece35497-bib-0067] Lopez-Perea, J. J. , & Mateo, R. (2019). Wax esters of uropygial gland secreation as biomarkers of endocrine disruption in birds exposed to treated sewage water. Environmental Polluation, 250, 323–330. 10.1016/j.envpol.2019.04.039 31003144

[ece35497-bib-0068] Matessi, G. , Carmagnani, C. , Griggio, M. , & Pilastro, A. (2008). Male rock sparrows differentially allocate nest defence but not food provisioning to offspring. Behaviour, 146, 209–223. 10.1163/156853909X410748

[ece35497-bib-0069] Mazerolle, M. J. (2017). Package ‘AICcmodavg’. R package.

[ece35497-bib-0070] McGlothlin, J. W. , Jawor, J. M. , Greives, T. J. , Casto, J. M. , Phillips, J. L. , & Ketterson, E. D. (2008). Hormones and honest signals: Males with larger ornaments elevate testosterone more when challenged. Journal of Evolutionary Biology, 21, 39–48. 10.1111/j.1420-9101.2007.01471.x 18034801

[ece35497-bib-0071] McLennan, D. , & Ryan, M. J. (1999). Interspecific recognition and discrimination based upon olfactory cues in northern swordtails. Evolution, 53(3), 880–888. 10.1111/j.1558-5646.1999.tb05382.x 28565635

[ece35497-bib-0072] McQuillan, M. A. , Huynh, A. V. , Taylor, S. A. , & Rice, A. M. (2017). Development of 10 novel SNP‐RFLP markers for quick genotyping within the black‐capped (*Poecile atricapillus*) and Carolina (*P. carolinensis*) chickadee hybrid zone. Conservation Genetics Resources, 9(2), 261–264. 10.1007/s12686-016-0667-z

[ece35497-bib-0073] McQuillan, M. A. , & Rice, A. M. (2015). Differential effects of climate and species interactions on range limits at a hybrid zone: Potential direct and indirect impacts of climate change. Ecology and Evolution, 5(21), 5120–5137. 10.1002/ece3.1774 26640687 PMC4662315

[ece35497-bib-0074] McQuillan, M. A. , Roth, T. C. , Huynh, A. V. , & Rice, A. M. (2018). Hybrid chickadees are deficient in learning and memory. Evolution, 72(5), 1155–1164. 10.1111/evo.13470 29578575

[ece35497-bib-0075] Mihailova, M. , Berg, M. L. , Buchanan, K. L. , & Bennett, A. T. D. (2014). Odour‐based discrimination of subspecies, species and sexes in an avian species complex, the crimson rosella. Animal Behaviour, 95, 155–164. 10.1016/j.anbehav.2014.07.012

[ece35497-bib-0076] Moller, A. P. , & Cuervo, J. J. (1998). Speciation and feather ornamentation in birds. Evolution, 52(3), 859–869. 10.1111/j.1558-5646.1998.tb03710.x 28565248

[ece35497-bib-0077] Nagel, L. , & Schluter, D. (1998). Body size, natural selection, and speciation in sticklebacks. Evolution, 52, 209–218. 10.1111/j.1558-5646.1998.tb05154.x 28568156

[ece35497-bib-0078] Odum, E. P. (1941). Annual cycle of the black‐capped chickadee. The Auk, 58(3), 314–333.

[ece35497-bib-0079] Olsson, S. B. , Linn, C. E. , Michel, A. , Dambroski, H. R. , Berlocher, S. H. , Feder, J. L. , & Roelofs, W. L. (2006). Receptor expression and sympatric speciation: Unique olfactory receptor neuron responses in F1 hybrid *Rhagoletis* populations. Journal of Experimental Biology, 209(19), 3729–3741. 10.1242/jeb.02444 16985190

[ece35497-bib-0080] Otter, K. , & Ratcliffe, L. (1996). Female initiated divorce in a monogamous songbird: abandoning mates for males of higher quality. Proceedings of the Royal Society B: Biological Sciences, 263(1368), 351–355.

[ece35497-bib-0081] Patten, M. A. , Rotenberry, J. T. , & Zuk, M. (2004). Habitat selection, acoustic adaptation, and the evolution of reproductive isolation. Evolution, 58(10), 2144–2155. 10.1111/j.0014-3820.2004.tb01593.x 15562681

[ece35497-bib-0082] Pfennig, D. W. , & Pfennig, K. S. (2012). Evolution's wedge: Competition and the origins of diversity. Berkeley, CA: University of California Press.

[ece35497-bib-0083] Podos, J. (2001). Correlated evolution of morphology and vocal signal structure in Darwin's finches. Nature, 409, 185–188. 10.1038/35051570 11196640

[ece35497-bib-0084] R Core Team (2018). R: A language and environment for statistical computing. Vienna, Austria: R Foundation for Statistical Computing Retrieved from https://ww.R-project.org/

[ece35497-bib-0085] Rafferty, N. E. , & Boughman, J. W. (2006). Olfactory mate recognition in a sympatric species pair of three‐spined sticklebacks. Behavioral Ecology, 17(6), 965–970. 10.1093/beheco/arl030

[ece35497-bib-0086] Reneerkens, J. , Piersma, T. , & Damsté, J. S. S. (2002). Sandpipers (Scolopacidae) switch from monoester to diester preen waxes during courtship and incubation, but why? Proceedings of the Royal Society of London. Series B: Biological Sciences, 269, 2135–2139. 10.1098/rspb.2002.2132 12396488 PMC1691136

[ece35497-bib-0087] Reneerkens, J. , Piersma, T. , & Damsté, J. S. S. (2005). Switch to diester preen waxes may reduce avian nest predation by mammalian predators using olfactory cues. The Journal of Experimental Biology, 208, 4199–4202. 10.1242/jeb.01872 16272242

[ece35497-bib-0088] Reynolds, R. G. , & Fitzpatrick, B. M. (2007). Assortative mating in poison‐dart frogs based on an ecologically important trait. Evolution, 61, 2253–2259. 10.1111/j.1558-5646.2007.00174.x 17767594

[ece35497-bib-0089] Robbins, M. B. , Braun, M. J. , & Tobey, E. A. (1986). Morphological and vocal variation across a contact zone between the chickadees *Parus atricapillus* and *P*. carolinensis. The Auk, 103(4), 655–666.

[ece35497-bib-0090] Ronald, K. L. , Fernández‐Juricic, E. , & Lucas, J. R. (2018). Mate choice in the eye and ear of the beholder? Female multimodal sensory configuration influences her preferences. Proceedings of the Royal Society B: Biological Sciences, 285, 20180713 10.1098/rspb.2018.0713 PMC5966616 29769366

[ece35497-bib-0091] Rossi, M. , Reinaldo, M. , Golüke, S. , Komdeur, J. , Korsten, P. , & Caspers, B. A. (2017). Begging blue tit nestlings discriminate between the odour of familiar and unfamiliar conspecifics. Functional Ecology, 31, 1761–1769. 10.1111/1365-2435.12886

[ece35497-bib-0092] Roth, T. C. , Cox, J. G. , & Lima, S. L. (2008). Can foraging birds assess predation risk by scent? Animal Behaviour, 76, 2021–2027.

[ece35497-bib-0093] Sætre, G. , Moum, T. , Bures, S. , Kral, M. , Adamjan, M. , & Moreno, J. (1997). A sexually selected character displacement in flycatchers reinforces premating isolation. Nature, 387, 1995–1998. 10.1038/42451

[ece35497-bib-0094] Sandilands, V. , Powell, K. , Keeling, L. , & Savory, C. J. (2004). Preen gland function in layer fowls: Factors affecting preen oil fatty acid composition. British Poultry Science, 45(1), 109–115. 10.1080/00071660410001668932 15115208

[ece35497-bib-0095] Sasakawa, K. , & Kon, Y. (2018). Learning‐induced host preference in male parasitoid wasps as a potential driver of ecological speciation. Journal of Evolutionary Biology, 31, 1750–1755. 10.1111/jeb.13363 30084139

[ece35497-bib-0096] Schaal, B. , & Orgeur, P. (1992). Olfaction in utero: Can the rodent model be generalized? Quarterly Journal of Experimental Psychology B, 44(3–4), 245–278. 10.1080/02724999208250615 1598422

[ece35497-bib-0097] Servedio, M. R. (2007). Male versus female mate choice: Sexual selection and the evolution of species recognition via reinforcement. Evolution, 61(12), 2772–2789. 10.1111/j.1558-5646.2007.00247.x 17924955

[ece35497-bib-0098] Servedio, M. R. , & Noor, M. A. F. (2003). The role of reinforcement in speciation: Theory and data. Annual Review of Ecology, Evolution, and Systematics, 34, 339–364. 10.1146/annurev.ecolsys.34.011802.132412

[ece35497-bib-0099] Servedio, M. R. , Van Doorn, G. S. , Kopp, M. , Frame, A. M. , & Nosil, P. (2011). Magic traits in speciation: ‘magic’ but not rare? Trends in Ecology and Evolution, 26(8), 389–397. 10.1016/j.tree.2011.04.005 21592615

[ece35497-bib-0100] Shahandeh, M. P. , Pischedda, A. , & Turner, T. L. (2017). Male mate choice via cuticular hydrocarbon pheromones drives reproductive isolation between *Drosophila* species. Evolution, 72(1), 123–135. 29098691 10.1111/evo.13389 PMC5760347

[ece35497-bib-0101] Shaw, C. L. , Rutter, J. E. , Austin, A. L. , Garvin, M. C. , & Whelan, R. J. (2011). Volatile and semivolatile compounds in gray catbird uropygial secretions vary with age and between breeding and wintering grounds. Journal of Chemical Ecology, 37(4), 329–339. 10.1007/s10886-011-9931-6 21424249

[ece35497-bib-0102] Singer, T. L. (1998). Roles of hydrocarbons in the recognition systems of insects. American Zoologist, 38, 394–405. 10.1093/icb/38.2.394

[ece35497-bib-0103] Slabbekoorn, H. , & Smith, T. B. (2002). Bird song, ecology and speciation. Philosophical Transactions of the Royal Society of London. Series B: Biological Sciences, 356, 493–503. 10.1098/rstb.2001.1056 PMC1692962 12028787

[ece35497-bib-0104] Smadja, C. , & Butlin, R. K. (2009). On the scent of speciation: The chemosensory system and its role in premating isolation. Heredity, 102, 77–97. 10.1038/hdy.2008.55 18685572

[ece35497-bib-0105] Sneddon, H. , Hadden, R. , & Hepper, P. G. (1998). Chemosensory learning in the chicken embryo. Physiology and Behavior, 64(2), 133–139. 10.1016/S0031-9384(98)00037-7 9662076

[ece35497-bib-0106] Snowberg, L. K. , & Bolnick, D. I. (2008). Assortative mating by diet in a phenotypically unimodal but ecologically variable population of stickleback. The American Naturalist, 172, 733–739. 10.1086/591692 18834291

[ece35497-bib-0107] Soini, H. A. , Schrock, S. E. , Bruce, K. E. , Wiesler, D. , Ketterson, E. D. , & Novotny, M. V. (2007). Seasonal variation in volatile compound profiles of preen gland secretions of the dark‐eyed junco (*Junco Hyemalis*). Journal of Chemical Ecology, 33(1), 183–198. 10.1007/s10886-006-9210-0 17146717

[ece35497-bib-0108] Soini, H. A. , Whittaker, D. J. , Wiesler, D. , Ketterson, E. D. , & Novotny, M. V. (2013). Chemosignaling diversity in songbirds: Chromatographic profiling of preen oil volatiles in different species. Journal of Chromatography A, 1317, 186–192. 10.1016/j.chroma.2013.08.006 23998336

[ece35497-bib-0109] Sonnenberg, B. R. , Branch, C. L. , Pitera, A. M. , Bridge, E. , & Pravosudov, V. V. (2019). Natural selection and spatial cognition in wild food‐caching mountain chickadees. Current Biology, 29(4), 670–676. 10.1016/j.cub.2019.01.006 30744977

[ece35497-bib-0110] Steiger, S. S. , Fidler, A. E. , Valcu, M. , & Kempenaers, B. (2008). Avian olfactory receptor gene repertoires: evidence for a well‐developed sense of smell in birds? Proceedings of the Royal Society B: Biological Sciences, 275(1649), 2309–2317. 10.1098/rspb.2008.0607 PMC2495045 18628122

[ece35497-bib-0111] Steiger, S. S. , Kuryshev, V. Y. , Stensmyr, M. C. , Kempenaers, B. , & Mueller, J. C. (2009). A comparison of reptilian and avian olfactory receptor gene repertoires: Species‐specific expansion of group γ genes in birds. BMC Genomics, 10, 446 10.1186/1471-2164-10-446 19772566 PMC2758906

[ece35497-bib-0112] Sun, L. , & Muller‐Schwarze, D. (1998a). Anal gland secretion codes for family membership in the beaver. Behavioral Ecology and Sociobiology, 44, 199–208. 10.1007/s002650050532

[ece35497-bib-0113] Sun, L. , & Muller‐Schwarze, D. (1998b). Anal gland secretion codes for relatedness in the beaver, Castor canadensis. Ethology, 104, 917–927.

[ece35497-bib-0114] Taylor, S. A. , Curry, R. L. , White, T. A. , Ferretti, V. , & Lovette, I. (2014). Spatiotemporally consistent genomic signatures of reproductive isolation in a moving hybrid zone. Evolution, 68(11), 3066–3081. 10.1111/evo.12510 25138643

[ece35497-bib-0115] Taylor, S. A. , White, T. A. , Hochachka, W. M. , Ferretti, V. , Curry, R. L. , & Lovette, I. (2014). Climate‐mediated movement of an avian hybrid zone. Current Biology, 24(6), 671–676. 10.1016/j.cub.2014.01.069 24613306

[ece35497-bib-0116] Tuttle, E. M. , Sebastian, P. J. , Posto, A. L. , Soini, H. A. , Novotny, M. V. , & Gonser, R. A. (2014). Variation in preen oil composition pertaining to season, sex, and genotype in the polymorphic white‐throated sparrow. Journal of Chemical Ecology, 40, 1025–1038. 10.1007/s10886-014-0493-2 25236380

[ece35497-bib-0117] Velando, A. , Beamonte‐Barrientos, R. , & Torres, R. (2006). Pigment‐based skin colour in the blue‐footed booby: An honest signal of current condition used by females to adjust reproductive investment. Oecologia, 149(3), 535–542. 10.1007/s00442-006-0457-5 16821015

[ece35497-bib-0118] Verrell, P. A. (1985). Male mate choice for large, fecund females in the red‐spotted newt, *Notophthalmus viridescens*: How is size assessed? Herpetologica, 41(4), 382–386.

[ece35497-bib-0119] Verzijden, M. N. , Cate, C. , Servedio, M. R. , Kozak, G. M. , Boughman, J. W. , & Svensson, E. I. (2012). The impact of learning on sexual selection and speciation. Trends in Ecology & Evolution, 27(9), 511–519. 10.1016/j.tree.2012.05.007 22705159

[ece35497-bib-0120] Wang, H. L. , Zhao, C. H. , & Wang, C. Z. (2005). Comparative study of sex pheromone composition and biosynthesis in *Helicoverpa armigera*, *H. assulta* and their hybrid. Insect Biochemistry and Molecular Biology, 35(6), 575–583. 10.1016/j.ibmb.2005.01.018 15857763

[ece35497-bib-0121] Warton, D. I. , & Hui, F. K. C. (2011). The arcsine is asinine: The analysis of proportions in ecology. Ecology, 92(1), 3–10. 10.1890/10-0340.1 21560670

[ece35497-bib-0122] Whelan, R. J. , Levin, T. C. , Owen, J. C. , & Garvin, M. C. (2010). Short‐chain carboxylic acids from gray catbird (*Dumetella carolinensis*) uropygial secretions vary with testosterone levels and photoperiod. Comparative Biochemistry and Physiology Part B: Biochemistry and Molecular Biology, 156(3), 183–188. 10.1016/j.cbpb.2010.03.005 20346408

[ece35497-bib-0123] Whittaker, D. J. , & Gerlach, N. M. (2016). Mate choice in dark‐eyed juncos using visual, acoustic, and chemical cues In Ketterson E. D., & Atwell J. W. (Eds.), Snowbird: Integrative biology and evolutionary diversity in the junco (pp. 281–309). Chicago, IL: University of Chicago Press.

[ece35497-bib-0124] Whittaker, D. J. , Gerlach, N. M. , Soini, H. A. , Novotny, M. V. , & Ketterson, E. D. (2013). Bird odour predicts reproductive success. Animal Behaviour, 86(4), 697–703. 10.1016/j.anbehav.2013.07.025

[ece35497-bib-0125] Whittaker, D. J. , Kuzel, M. , Burrell, M. J. E. , Soini, H. A. , Novotny, M. V. , & DuVal, E. H. (2019). Chemical profiles reflect heterozygosity and seasonality in a tropical lekking passerine bird. Animal Behaviour, 151, 67–75. 10.1016/j.anbehav.2019.03.005

[ece35497-bib-0126] Whittaker, D. J. , Richmond, K. M. , Miller, A. K. , Kiley, R. , Burns, C. B. , Atwell, J. W. , & Ketterson, E. D. (2011). Intraspecific preen oil odor preferences in dark‐eyed juncos (*Junco hyemalis*). Behavioral Ecology, 22(6), 1256–1263. 10.1093/beheco/arr122

[ece35497-bib-0127] Whittaker, D. J. , Rosvall, K. A. , Slowinski, S. P. , Soini, H. A. , Novotny, M. V. , & Ketterson, E. D. (2018). Songbird chemical signals reflect uropygial gland androgen sensitivity and predict aggression: Implications for the role of the periphery in chemosignaling. Journal of Comparative Physiology A, 204(1), 5–15. 10.1007/s00359-017-1221-5 29063285

[ece35497-bib-0128] Whittaker, D. J. , Soini, H. A. , Atwell, J. W. , Hollars, C. , Novotny, M. V. , & Ketterson, E. D. (2010). Songbird chemosignals: Volatile compounds in preen gland secretions vary among individuals, sexes, and populations. Behavioral Ecology, 21(3), 608–614. 10.1093/beheco/arq033 22475692 PMC2854530

[ece35497-bib-0129] Yang, Y. , Blomenkamp, S. , Dugas, M. B. , Richards‐Zawacki, C. L. , & Pröhl, H. (2019). Mate choice versus mate preference: Inferences about color‐assortative mating differ between field and lab assays of poison frog behavior. The American Naturalist, 193(4). 10.1086/702249 30912970

[ece35497-bib-0130] Yang, Y. , Richards‐Zawacki, C. L. , Devar, A. , & Dugas, M. B. (2016). Poison frog color morphs express assortative mate preferences in allopatry but not sympatry. Evolution, 70(12), 2778–2788. 10.1111/evo.13079 27704539

[ece35497-bib-0131] Zhang, J. , Sun, L. , & Zuo, M. (2009). Uropygial gland volatiles may code for olfactory information about sex, individual, and species in Bengalese finches *Lonchura striata* . Current Zoology, 55(5), 357–365.

[ece35497-bib-0132] Zhang, J. , Wei, W. , Zhang, J. , & Yang, W. (2010). Uropygial gland‐secreted alkanols contribute to olfactory sex signals in budgerigars. Chemical Senses, 35(5), 375–382. 10.1093/chemse/bjq025 20212012

[ece35497-bib-0133] Zhang, Y. , Du, Y. , & Zhang, J. (2013). Uropygial gland volatiles facilitate species recognition between two sympatric sibling bird species. Behavioral Ecology, 24(6), 1271–1278. 10.1093/beheco/art068

