# Supplementary material for: Conspecific olfactory preferences and interspecific divergence in odor cues in a chickadee hybrid zone
Source: Ecol Evol. 2019 Aug 1;9(17):9671–83. doi: 10.1002/ece3.5497 (PMC6745874; doi:10.1002/ece3.5497)
Supplement: Supplementary file 1 [file ECE3-9-9671-s001.docx]

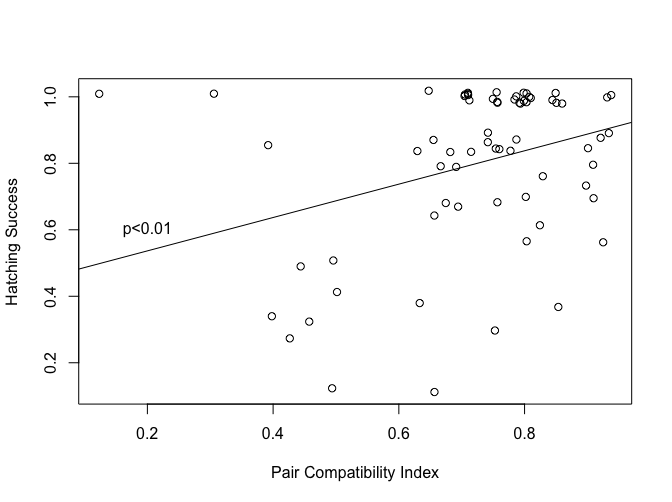


Figure S1: Hatching data from nests within artificial PVC housing was collected during the spring from 2013 to 2016 (n=68) in our eastern Pennsylvania study populations. The compatibility index for each nest was calculated based on the proportion of homozygous offspring those parents could produce at 10 SNP loci (see methods), averaged across all loci (Bronson et al. 2005). Linear regression of the proportion of eggs that successfully hatched by compatibility index values shows a significant relationship (p<0.01).


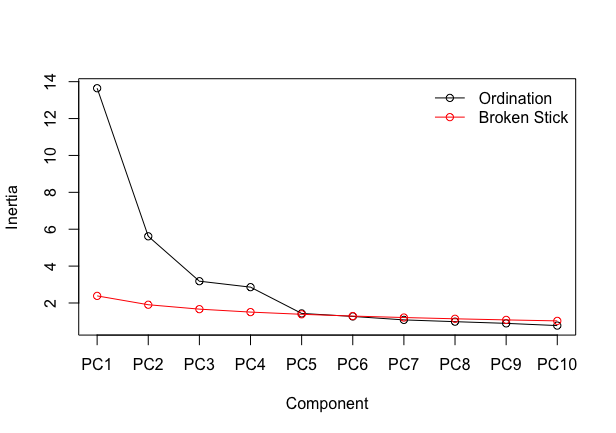


Figure S2: Scree plot of the first 10 principal components in black with a broken-stick distribution overlaid in red.


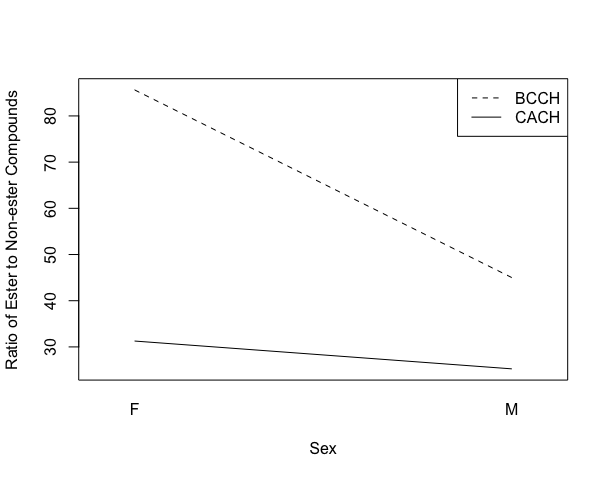


Figure S3: Interaction plot of the ratio of ester to non-ester compounds by species and sex.


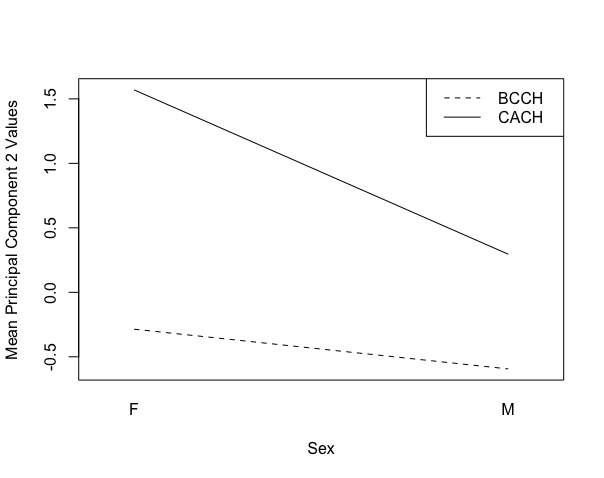


Figure S4: Interaction plot of PC2 by species and sex.


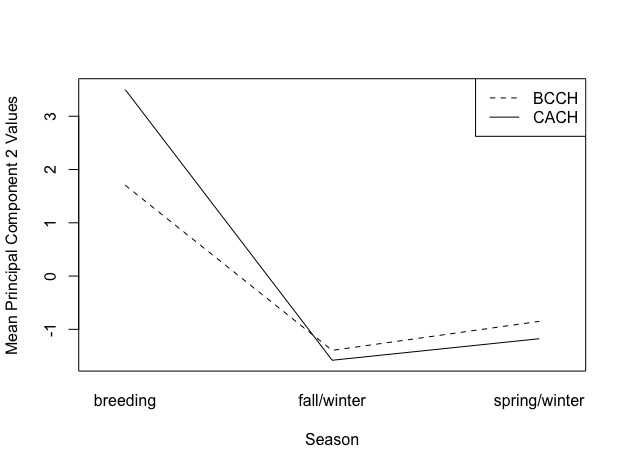


Figure S5: Interaction plot of PC2 by species and season.


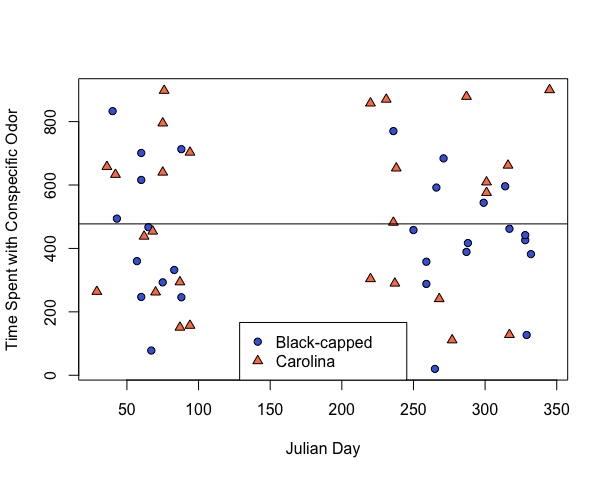


Figure S6: Time spent with conspecific odors in Y-maze trials throughout the year. No statistical effect of species, sex, or date on conspecific preference (Adjusted R^2^ = -0.03, F_7,47_ = 0.79, P = 0.69).

| **Peaks** | **Rotation.PC1** | **Rotation.PC2** |
| --- | --- | --- |
| ret.3.5 | -0.0005233 | -0.0036486 |
| ret.4.3 | -0.1008268 | -0.4726376 |
| ret.4.8 | -0.0069665 | -0.0115918 |
| ret.5.3 | -0.0411485 | -0.1470046 |
| ret.5.5 | -0.3468043 | 0.42045712 |
| ret.6.1 | -0.0283988 | -0.1002212 |
| ret.6.4 | -7.93E-05 | -0.0003549 |
| ret.6.7 | -0.019284 | -0.0712695 |
| ret.7.1 | -0.0095732 | 0.01212137 |
| ret.7.3 | -0.0324778 | 0.07413373 |
| ret.7.5 | -0.0973597 | 0.05184852 |
| ret.7.7 | -0.0053893 | -0.0134979 |
| ret.7.9 | -0.0005636 | -0.0023029 |
| ret.8.3 | -0.0258645 | -0.080681 |
| ret.8.4 | -0.0097515 | -0.0221627 |
| ret.8.6 | -0.0129191 | -0.0312656 |
| ret.8.9 | -0.0186916 | -0.0515149 |
| ret.9.1 | -0.0032888 | -0.0100248 |
| ret.9.4 | -0.0603538 | -0.0097208 |
| ret.9.5 | -0.0022372 | -0.0097267 |
| ret.9.6 | -0.0015731 | -0.0071488 |
| ret.9.8 | -0.0720335 | 0.02766275 |
| ret.9.9 | -0.0137213 | -0.0408182 |
| ret.10.0 | -0.016379 | -0.0470206 |
| ret.10.5 | -0.1921971 | 0.09191328 |
| ret.10.6 | -0.1734777 | 0.15512083 |
| ret.10.8 | -0.0953828 | 0.04194792 |
| ret.11.0 | -0.0020524 | -0.0064355 |
| ret.11.2 | -0.0190409 | -0.0490614 |
| ret.11.4 | -0.0081625 | -0.0213363 |
| ret.11.6 | -0.0644959 | 0.04293005 |
| ret.11.9 | 0.00094102 | -0.0004128 |
| ret.12.3 | -0.0200103 | -0.0245187 |
| ret.12.6 | -0.0501385 | -0.0173324 |
| ret.12.7 | -0.0069424 | -0.0272367 |
| ret.13.0 | 0.00067968 | -0.0035113 |
| ret.13.3 | -0.0138958 | -0.0300378 |
| ret.13.6 | -0.0104398 | -0.0359583 |
| ret.13.7 | -0.0226667 | -0.0713845 |
| ret.13.9 | -0.0043736 | -0.010923 |
| ret.14.0 | -0.0005662 | -0.0024009 |
| ret.14.2 | -0.0069533 | -0.0181108 |
| ret.14.4 | -0.0156321 | -0.0566761 |
| ret.14.6 | -0.1713736 | 0.08285899 |
| ret.14.7 | -0.0030872 | -0.0083865 |
| ret.14.9 | -0.0131103 | -0.0393355 |
| ret.15.3 | -0.0113397 | -0.0288444 |
| ret.15.4 | -0.0236233 | -0.0644772 |
| ret.15.6 | -0.1270634 | 0.07084842 |
| ret.15.8 | -0.0329376 | -0.0347314 |
| ret.16.1 | -0.0316993 | -0.0393578 |
| ret.16.3 | -0.0074568 | -0.0218349 |
| ret.16.5 | -0.0014636 | -0.0024354 |
| ret.17.0 | -0.0074121 | -0.056946 |
| ret.17.1 | -0.0113364 | -0.0397654 |
| ret.17.4 | -0.002616 | -0.0082654 |
| ret.17.6 | -0.0112238 | -0.0414889 |
| ret.17.8 | -0.016469 | -0.0529256 |
| ret.18.2 | -0.0100984 | -0.0355012 |
| ret.18.6 | -0.0116093 | -0.0356834 |
| ret.18.9 | -0.0095181 | -0.0309624 |
| ret.19.0 | -0.0028619 | -0.0067481 |
| ret.19.1 | -0.01822 | -0.0689234 |
| ret.19.5 | -0.0173229 | -0.0912398 |
| ret.19.6 | -0.0087452 | -0.027333 |
| ret.19.7 | -0.0009088 | -0.0037279 |
| ret.20.0 | -0.0655536 | 0.03912932 |
| ret.20.2 | -0.0091858 | -0.0283794 |
| ret.20.4 | -0.0156211 | -0.0370929 |
| ret.20.5 | -0.0160028 | -0.0474121 |
| ret.20.7 | -0.0095811 | -0.0265408 |
| ret.21.1 | -0.0128483 | -0.0085219 |
| ret.21.3 | -0.0179612 | -0.0635803 |
| ret.21.6 | -0.0057624 | -0.0189556 |
| ret.22.2 | -0.0112467 | -0.0428705 |
| ret.22.6 | -0.0072392 | -0.0258482 |
| ret.23.2 | -0.0242262 | -0.0004226 |
| ret.23.4 | -0.0129173 | -0.0487932 |
| ret.23.7 | -0.0156595 | -0.0598975 |
| ret.23.9 | -0.0283896 | 0.02965796 |
| ret.24.4 | -0.0125327 | -0.0751182 |
| ret.24.7 | -0.0003103 | 0.01885109 |
| ret.25.0 | -0.0014819 | -0.0032571 |
| ret.25.3 | -0.0054628 | -0.0304107 |
| ret.25.6 | -0.0061375 | -0.0239878 |
| ret.26.4 | -0.0020084 | -0.0124839 |
| ret.26.8 | -0.0269592 | -0.1188998 |
| ret.27.1 | -0.013309 | 0.00826394 |
| ret.27.7 | -0.0061627 | -0.0330291 |
| ret.27.9 | -0.007658 | 0.00664991 |
| ret.28.4 | -0.0115741 | -0.0825549 |
| ret.28.6 | 0.00060043 | 0.00465951 |
| ret.29.3 | 1.62E-05 | -0.0017951 |
| ret.30.0 | -0.0105771 | -0.0002696 |
| ret.30.4 | -0.0021379 | -0.0216443 |
| ret.31.1 | 0.00102486 | -0.0056929 |
| ret.31.5 | -0.0365575 | -0.2078672 |
| ret.32.2 | 0.00075597 | 0.00052103 |
| ret.33.0 | 0.00318129 | -0.0037749 |
| ret.33.5 | -0.0008322 | -0.0062562 |
| ret.34.0 | 0.00029001 | -0.0016284 |
| ret.34.5 | 0.00488738 | -0.0035577 |
| ret.35.0 | -0.020814 | -0.028074 |
| ret.35.2 | 0.00211706 | 0.00088349 |
| ret.36.0 | 0.03193728 | 0.0287947 |
| ret.36.5 | 0.00522292 | 0.00293799 |
| ret.36.6 | 0.00053294 | 0.00025672 |
| ret.37.0 | 0.00788704 | 0.00842619 |
| ret.37.4 | 0.04009496 | 0.03376733 |
| ret.37.9 | -0.0036367 | -0.0872008 |
| ret.38.4 | 0.00778112 | -0.0289409 |
| ret.38.8 | 0.1475862 | 0.14264543 |
| ret.39.2 | 0.06770534 | 0.06255159 |
| ret.39.4 | 0.00436744 | 0.00234536 |
| ret.39.7 | 0.03002887 | 0.0553179 |
| ret.40.1 | 0.14028848 | 0.09921515 |
| ret.40.55 | 0.07646964 | 0.05664816 |
| ret.41.1 | 0.02144312 | 0.02064534 |
| ret.41.4 | 0.26677344 | 0.1515481 |
| ret.41.8 | 0.16543314 | 0.09982172 |
| ret.41.95 | 0.00054741 | -0.0012658 |
| ret.42.3 | 0.05960957 | 0.03943728 |
| ret.42.7 | 0.21768614 | 0.12717251 |
| ret.42.8 | 0.0227706 | 0.02043989 |
| ret.43.1 | 0.15947484 | 0.09875863 |
| ret.43.6 | 0.02646072 | 0.00865477 |
| ret.43.9 | 0.31428211 | 0.16599695 |
| ret.44.4 | 0.24658343 | 0.10771112 |
| ret.44.5 | 0.00991441 | -0.0140147 |
| ret.45.0 | 0.05658791 | -0.0201003 |
| ret.45.4 | 0.219656 | -0.0238461 |
| ret.46.0 | 0.19267521 | 0.03670801 |
| ret.46.7 | 0.01934866 | -0.0567213 |
| ret.47.3 | 0.28503346 | -0.001464 |
| ret.47.9 | 0.2303397 | -0.0144743 |
| ret.48.1 | 0.02948442 | 0.02456905 |
| ret.48.8 | 0.03982386 | -0.0608857 |
| ret.49.4 | 0.14408667 | -0.0715452 |
| ret.49.9 | 0.00476538 | -0.0379999 |
| ret.50.2 | 0.12114823 | -0.0469941 |
| ret.51.4 | -0.0031663 | -0.1602427 |
| ret.52.1 | 0.15685462 | -0.161997 |
| ret.53.1 | 0.12776508 | -0.2525745 |
| ret.54.3 | 0.02049768 | -0.0877553 |
| ret.55.5 | 0.05219239 | -0.1742064 |
| ret.56.5 | 0.05033196 | -0.140035 |

Table S1: Variable loading values of principal components 1 and 2. Peaks are identified by their elution time (see methods).
